# Salinity and temperature significantly influence seed germination, seedling establishment, and seedling growth of eelgrass *Zostera marina* L.

**DOI:** 10.7717/peerj.2697

**Published:** 2016-11-15

**Authors:** Shaochun Xu, Yi Zhou, Pengmei Wang, Feng Wang, Xiaomei Zhang, Ruiting Gu

**Affiliations:** 1 Key Laboratory of Marine Ecology & Environmental sciences, Institute of Oceanology, Chinese Academy of Sciences, Qingdao, China; 2 Department of Bioscience, University of Chinese Academy of Sciences, Beijing, China; 3 Laboratory for Marine Ecology and Environmental Science, Qingdao National Laboratory for Marine Science and Technology, Qingdao, China

**Keywords:** *Zostera marina* L., Salinity, Temperature, Seed germination, Seedling establishment, Seedling growth, Seagrass

## Abstract

Globally, seagrass beds have been recognized as critical yet declining coastal habitats. To mitigate seagrass losses, seagrass restorations have been conducted in worldwide over the past two decades. Seed utilization is considered to be an important approach in seagrass restoration efforts. In this study, we investigated the effects of salinity and temperature on seed germination, seedling establishment, and seedling growth of eelgrass *Zostera marina* L. (Swan Lake, northern China). We initially tested the effects of salinity (0, 5, 10, 15, 20, 25, 30, 35, and 40 ppt) and water temperature (5, 10, 15, and 20 °C) on seed germination to identify optimal levels. To identify levels of salinity that could potentially limit survival and growth, and, consequently, the spatial distribution of seedlings in temperate estuaries, we then examined the effect of freshwater and other salinity levels (10, 20, and 30 ppt) on seedling growth and establishment to confirm suitable conditions for seedling development. Finally, we examined the effect of transferring germinated seeds from freshwater or low salinity levels (1, 5, and 15 ppt) to natural seawater (32 ppt) on seedling establishment rate (SER) at 15 °C. In our research, we found that: (1) Mature seeds had a considerably lower moisture content than immature seeds; therefore, moisture content may be a potential indicator of *Z. marina* seed maturity; (2) Seed germination significantly increased at low salinity (p < 0.001) and high temperature (p < 0.001). Salinity had a much stronger influence on seed germination than temperature. Maximum seed germination (88.67 ± 5.77%) was recorded in freshwater at 15 °C; (3) Freshwater and low salinity levels (< 20 ppt) increased germination but had a strong negative effect on seedling morphology (number of leaves per seedling reduced from 2 to 0, and maximum seedling leaf length reduced from 4.48 to 0 cm) and growth (seedling biomass reduced by 46.15–66.67% and maximum seedling length reduced by 21.16–69.50%). However, *Z. marina* performed almost equally well at salinities of 20 and 30 ppt. Very few germinated seeds completed leaf differentiation and seedling establishment in freshwater or at low salinity, implying that freshwater and low salinity may potentially limit the distribution of this species in coastal and estuarine waters. Therefore, the optimum salinity for *Z. marina* seedling establishment and colonization appears to be above 20 ppt in natural beds; (4) Seeds germinated in freshwater or at low salinity levels could be transferred to natural seawater to accomplish seedling establishment and colonization. This may be the optimal method for the adoption of seed utilization in seagrass restoration. We also identified seven stages of seed germination and seedling metamorphosis in order to characterize growth and developmental characteristics. Our results may serve as useful information for *Z. marina* habitat establishment and restoration programs.

## Introduction

Seagrass meadows, together with mangroves and coral reefs, are extremely valuable coastal marine ecosystems that are widely acknowledged for both their ecological and economic importance. They provide essential habitat and nurseries for a variety of marine organisms, and play critical roles in primary and secondary production, as well as coastal ecosystem nutrient cycles (Costanza et al., 1997; Hemminga & Carlos, 2000; Ogden, 2006; Orth et al., 2006a; Liu et al., 2013; Connolly & Waltham, 2015; McCloskey & Unsworth, 2015; Tol, Coles & Congdon, 2016). However, seagrass meadows are declining globally (Short et al., 2011) due to anthropogenic activities (e.g. port infrastructure development and dredging) and natural disturbances (e.g. disease and storms) (Cambridge & McComb, 1984; Short & Burdick, 1996; Wyllie-Echeverria & Ackerman, 2003; Kim et al., 2015; Macreadie et al., 2015; Zhang et al., 2014; Lin et al., 2016; Govers et al., 2016). These issues have led to the recent development of restoration efforts to compensate or mitigate seagrass losses, and to enhance the associated ecosystem services (Shafer & Bergstrom, 2010; Suykerbuyk et al., 2016).

In an effort to restore seagrass meadows on large-scales, a seagrass transplanting technique was developed to transplant mature or adult plants (Fonseca et al., 1994; Zhou et al., 2014; van Katwijk et al., 2016). However, such transplanting is highly labor- and cost-intensive, and harmful to the donor meadows, especially in a large-scale restoration project (Orth et al., 2000; Paling et al., 2009). Given these drawbacks, large scale seed broadcasting, evolved out of a long-term trialing process since the end of the last century, could be a relative efficient and cost-effective approach to restoring seagrass habitats. Seed broadcasting is one of the most promising approaches (Harwell & Orth, 1999; Orth et al., 2003; Olesen et al., 2004; Orth, Harwell & Lnglis, 2006; Shafer & Bergstrom, 2010); it has been successfully applied to a few seagrass restoration programs (Paling et al., 2009), such as the restoration of *Zostera marina* habitats in the United States (Orth et al., 2006b). Unfortunately, broadcast seeds have a very low germination rate in the field (compared with transplanted adult plants) because some seeds do not germinate or may be predated on by other animals leading to seed loss (Wassenberg, 1990; Orth, Luckenbach & Moore, 1994; Fishman & Orth, 1996; Orth et al., 2009). However, broadcasting germinated seeds and transplanting artificial seedlings are both important in seagrass restoration and could largely increase the seedling establishment rate (SER) (Wassenberg, 1990; Christensen, Sortkjaer & McGlathery, 1995; Fishman & Orth, 1996; Kirkman, 1999; Balestri, Piazzi & Cinelli, 1998; Orth et al., 2009).

Eelgrass (*Z. marina*) is the most widespread species throughout the temperate northern hemisphere of the Atlantic and Pacific (Green & Short, 2003; Olsen et al., 2016). Growth and physiology of adult plants have received considerable amounts of attention over the last three decades (Phillips, 1972; Phillips, McMillan & Bridges, 1983; Hemminga & Carlos, 2000; Touchette & Burkholder, 2000; Phillips, Milchakova & Alexandrov, 2006; Lee, Park & Kim, 2007; Leoni et al., 2008; Collier, Waycott & McKenzie, 2012; Dooley et al., 2013; Dooley et al., 2015; Kaldy et al., 2015); however, there is by far less information available concerning the key environmental factors, such as salinity and temperature, that influence seed germination, seedling establishment, and seedling growth (Orth & Moore, 1983; Hootsmans, Vermaat & Van Vierssen, 1987; Conacher, Poiner & O’Donohue, 1994; Abe, Kurashima & Maegawa, 2008; Tanner & Parham, 2010; Salo & Pedersen, 2014; Park, Lee & Son, 2014; Kaldy et al., 2015). Moreover, results to date have been inconsistent as to whether or not the salinity significantly effects seed germination, which might be influenced by the design of salinity levels (Orth et al., 2000). We hypothesized that salinity levels might significantly influence seed germination, seedling establishment, and seedling growth of *Z. marina*. In this study, we investigated the effects of salinity and temperature on seed germination, seedling establishment, and seedling growth of *Z. marina* L. (Swanlake Lagoon, northern China) in order to determine optimal conditions for this species. This study intends to provide fundamental information for creating adequate seed germination conditions and developing seedling establishment methods for eelgrass habitat establishment and restoration.

## Materials and Methods

### Ethic statement

The collecting of the reproductive shoots of *Z. marina* from Swan Lake of Weihai was permitted by Peiliang Wang, manager of Mashan Group Co. Ltd. Ethical approval was not required for this study because no endangered animals and plants were involved. However, specimen collection and maintenance were performed in strict accordance with the recommendations of China Society of Plant Protection.

### Seed collection

Between July 17 and 19, 2015, reproductive shoots of *Z. marina* with inflorescences containing developing or developed seeds were collected by hand from Swan Lake, a marine lagoon in the northeast of Rongcheng city, northern China (37°20′58.7″N, 122°34′26.9″E). Reproductive shoots were placed in coolers and transported to lab within 24 h of collection.

In the laboratory, reproductive shoots were stored in a 600 μm mesh bag, held in a circular, aerated flow-through tank (1 × 1.2 × 1.5 m) in natural sunlight. The mesh bag, containing reproductive shoots, was stirred by hand daily and held in the tank until the shoots degenerated and the seeds were released. Floating leaf material was removed, while seeds and plant materials were retained. Accumulated detritus was passed over a 2.5 mm sieve to remove large debris, and detritus material was separated from *Z. marina* seeds through a 1.0 mm sieve (Wyllie-Echeverria et al., 2003). Following this, all seeds were kept in high salinity artificial seawater (50 ppt) (Pan et al., 2011b) at 5 °C (Zarranz et al., 2010; Dooley, Wyllie-Echeverria & Van Volkenburgh, 2013) until initiation of our tests. With the maturation of seeds, the oyster white seeds (immature) become cyan, then brown or black (mature) (Pan et al., 2014). Only mature seeds in dark color were used in this experiment.

According to their color (oyster white, cyan, and black), seeds were classified as one of three degrees of maturity (immature, medium, and advanced maturity, respectively). To analyze the moisture content of *Z. marina* seeds at different degrees of maturity, 30 seeds were selected of each color and randomly divided to three replicates. Seeds were spread to dry on soft paper towels to remove water, and the wet weight (WW) of seeds was measured. Seeds were then placed in a drier for 72 h (60 °C), and their dry weights (DW) were measured. Moisture content percentage (MCP) of the *Z. marina* seeds was calculated using the equation:

(1) $${{\rm MCP} = {{\left({{\rm{WW-DW}}} \right)} \over {{\rm{DW}}}} \times {\rm{100\% }}}$$

### Seed germination rate at different salinities and temperatures

This experiment was initiated on November 3, 2015. Seeds used in this experiment were mature and black in color. Germination of seeds was explicitly defined as, not just the rupture of the seed coat, but also the emergence and growth of the cotyledon (Churchill, 1983; Brenchley & Probert, 1998). Seeds were placed in 12 cm plastic petri dishes containing 50 ml of freshwater or artificial seawater with different salinities (5, 10, 15, 20, 25, 30, 35, and 40 ppt) at 5, 10, 15, and 20 °C for a period of four weeks. Each treatment involved three replicate petri dishes containing 50 seeds each. Germinated seeds were counted after 4 weeks. Petri dishes were placed in an incubator, and artificial seawater was changed every 3 days.

### Seedling growth and establishment at different salinities at 5 °C

To examine the effect of salinity on seedling growth and establishment *Z. marina*, seeds and seedlings were cultured at different salinities (0, 10, 20 and 30 ppt) at 5 °C for six weeks. One hundred seeds were placed in 12 cm plastic Petri dishes containing 50 ml of freshwater or artificial seawater of different salinities (10, 20 and 30 ppt) at 5 °C, with three petri dishes per salinity treatment. Petri dishes were placed in an incubator, and artificial seawater was changed every three days, as described above. All the germinated seeds in each petri dish were transferred to each container with artificial seawater of corresponding salinity to continue culture. The seedling maximum length, weight, number of leaves, and maximum leaf length were measured in the container at the 3rd and 6th week. Any other morphological changes in *Z. marina* seeds and seedlings during and after germination were also recorded.

### Seedling establishment rate for seeds germinated at reduced salinities and transferred to natural seawater (32 ppt)

This experiment was conducted to examine the effects of transferring the germinated seeds from low salinity to natural seawater on seedling establishment. Fifty seeds were placed in 12 cm plastic petri dishes containing 50 ml of diluted seawater of different salinities (0, 1, 5, and 15 ppt) at 15 °C, with three petri dishes per salinity treatment. Petri dishes were placed in an incubator, and artificial seawater was changed every three days, as described above. Germinated seeds were immediately transferred to natural seawater (32 ppt) to continue culture at 15 °C. This is because prolonged exposure to freshwater is likely to result in increased incidence of rotten seeds and poor subsequent seedling survival (Kaldy et al., 2015). After four weeks, the number of seedlings from the germinated seeds was counted, and the SER was calculated using the equation:

(2) $${{\rm SER} = {{\rm{n}} \over {\rm{N}}}}$$

where n is the number of seedling from germinated seeds; N is the total number of seeds used for germination.

### Data analysis

A two-way analysis of variance (ANOVA) was used to compare the effects of temperature and salinity. When the interaction was significant, one-way ANOVA was used to compare the temperature effect at each salinity, and the salinity effect at each temperature (Zar, 1999). Moisture content of seeds, SER, and seedling maximum length, weight, and maximum leaf length were also analyzed by one-way ANOVA. The effect of salinity on seedling establishment was statistically analyzed by one-way ANOVA.

In the analysis, homogeneity of variance was tested using Levene’s test (Zar, 1999). When ANOVA identified a significant difference, the Tukey test was applied to identify specific treatment differences; when it has no significant difference, the Dunnett T3 test was applied to identify specific treatment differences. Analysis was based on the data collected in the experiment. R version 3.2.1 and SPSS 18.0 for Windows 8.1 were used for all data analyses. Differences were considered significant at a probability level of p < 0.05.

## Results

### Moisture content of seeds

*Zostera marina* seeds were classified by seed color (oyster white, cyan, or black), representing the various degrees of seed maturity (immature, medium, and advanced maturity, respectively). The moisture content and WW of seeds at different degrees of maturity both exhibited a significant difference (F = 1,055, df = 17, p < 0.001; F = 1,253, df = 17, p < 0.001, respectively). As shown in Table 1, the moisture content of seeds significantly decreased with increasing maturity; in contrast, the WW of seeds significantly increased with increasing maturity. Therefore, moisture content, together with seed color and weight, may be a potential indicator of *Z. marina* seed maturity. The average WW of *Z. marina* mature seeds selected for germination was 12.50 ± 0.04 mg, and the mean moisture content was 37.41 ± 0.74% (Table 1).

**Table 1:** Seed color, wet seed mass, moisture content, and maturity degree of *Zostera marina* L.

Seed color	Seed mass (mg)	Moisture content (%)	Degree of maturity
Oyster white	6.47 ± 0.24	58.42 ± 0.32^a^	Immature
Cyan	7.81 ± 0.12	40.02 ± 0.68^b^	Medium
Black	12.50 ± 0.04	37.41 ± 0.74^c^	Advanced maturity

**Note:**

Different letters indicate significant differences at p < 0.05 (mean ± standard error).

### Seed germination at different salinities and temperatures

Germination rates differed significantly (F = 53.713, df = 107, p < 0.001) at different temperatures (Table 2). Germination rates also differed significantly (F = 266.596, df = 107, p < 0.001) between different salinities. Seed germination rates were significantly higher at higher temperatures and seed germination rates decreased with increasing salinities. Temperature and salinity had significant interactive effects on germination rates (F = 3.634, df = 107, p < 0.001).

**Table 2:** Two-way analysis of variance of temperature and salinity on the germination rate of *Zostera marina* L. seeds.

Variable	Df	Sum Sq	Mean Sq	F value	P (> F)
Temperature	3	0.567	0.192	53.713	< 0.001
Salinity	8	7.623	0.9528	266.596	< 0.001
Temperature × salinity	24	0.312	0.013	3.634	< 0.001
Residuals	72	0.257	0.0036		

**Note:**

Data are not transformed and assumption of homogeneity of variance is met by Levene’s test (P = 0.3514).

**Figure 1:**
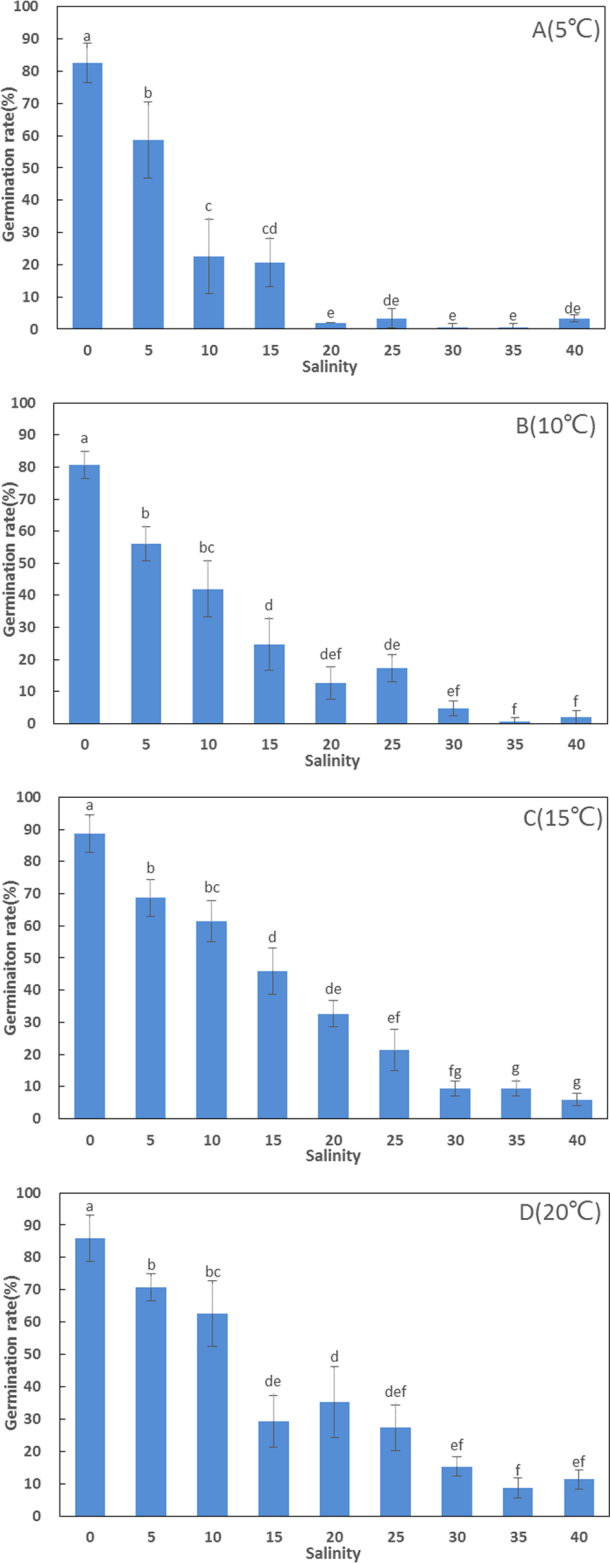
Germination rates (%) of *Zostera marina* L. seeds subjected to the different salinities and temperatures after four weeks. Different letters above error bars indicate significant differences at p < 0.05 (one-way analysis of variance) (mean ± standard error (SE), n = 3) (bars represent SE).

Germination rates (80.67–88.67%) in freshwater were significantly higher than in artificial seawater at high salinities (F = 82.094, df = 107, p < 0.001). There was no significant difference in germination rate between different temperatures in freshwater (F = 1.076, df = 11, p = 0.412). The highest germination rate (88.67 ± 5.77%) among all treatments was recorded at 15 °C in freshwater, and ANOVA detected significant differences between the remaining treatments at 15 °C (6–69%) at high salinities. At higher salinities (30, 35, and 40 ppt), germination rates were below 16%, but increased considerably when salinity decreased below 20 ppt (Fig. 1).

Generally, germination rates increased considerably with increasing temperature. Above 10 ppt, germination rates at 15 and 20 °C were significantly higher than at 5 °C (Fig. 1).

### Seedling growth and establishment at different salinities at 5 °C

**Figure 2:**
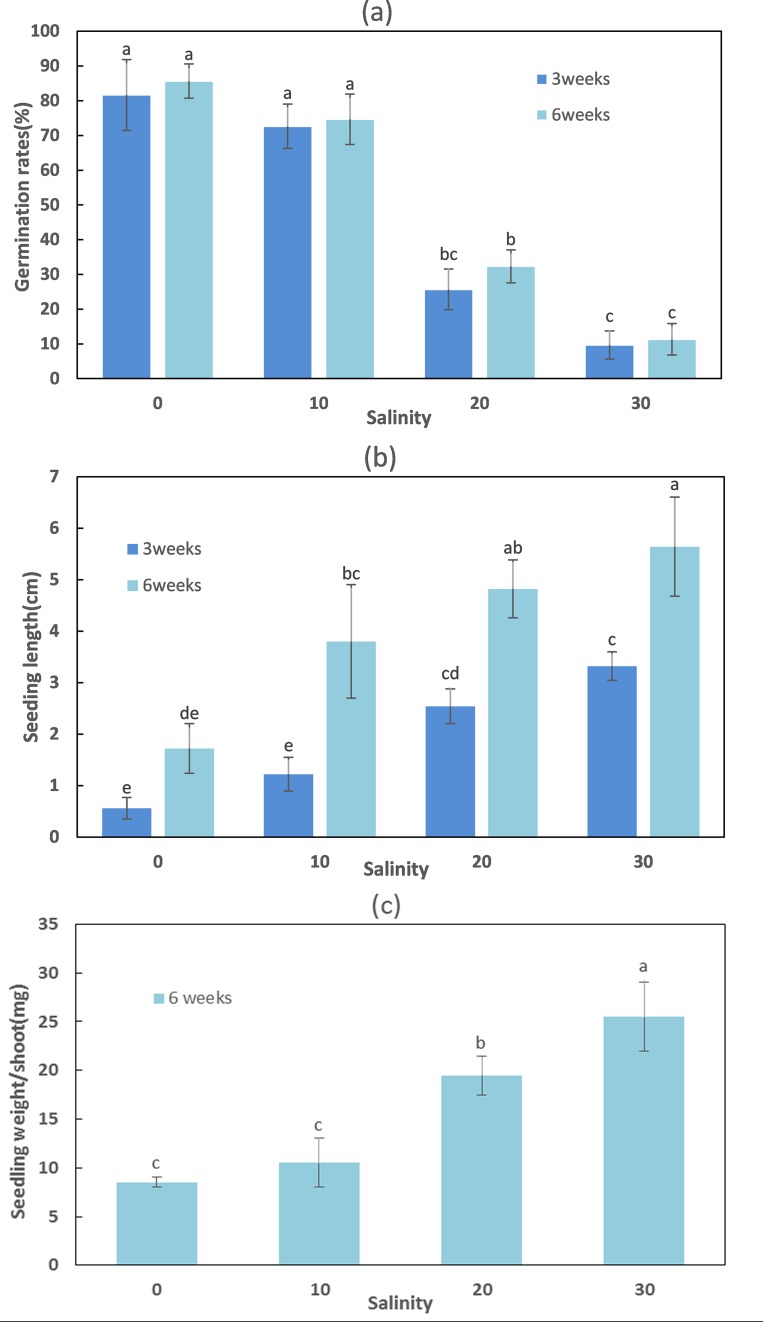
Seed germination, length, and weight of *Zostera marina* L. seedlings subjected to different salinities at 15 °C after three and six weeks. Different letters indicate significant differences at p < 0.05 (one-way ANOVA) (means ± SE, n = 3).

**Figure 3:**
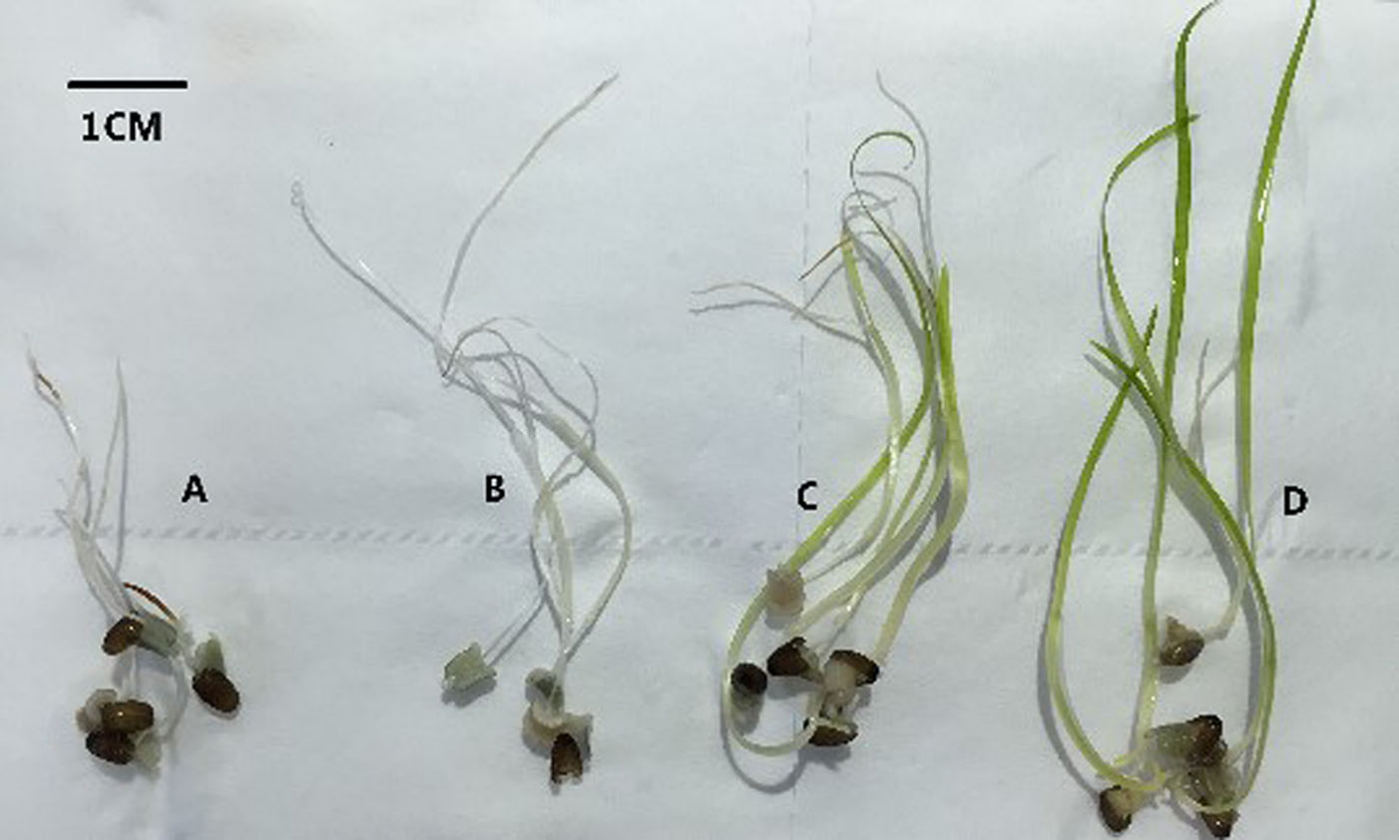
Morphology of *Zostera marina* L. seedlings after six weeks (bar represents 1 cm) at 5 °C. The germinated seeds were incubated in freshwater (A), 10 ppt (B), 20 ppt (C), and 30 ppt (D). (A) Germinated seeds with a cotyledon exceeding 1 cm; (B) Cotyledon exceeds 3 cm; (C) Leaf and adventitious root differentiation with the leaf length exceeding 4 cm; (D) Leaves exceed 5 cm in length.

Salinity appeared to play an important role in seedling establishment and growth (Figs. 2 and 3; Table 3). After six weeks, the highest germination rate (85.67 ± 4.93%) among all treatments was recorded in freshwater, consistent with the results of the seed germination experiment. The germination rates at lower salinities (0 and 10 ppt) were significantly higher than at higher salinities (20 and 30 ppt) (F = 94.376, df = 11, p < 0.001). The seedling maximum length, weight, number of leaves, and differentiation of *Z. marina* differed significantly (p < 0.001) between the lower salinities and the higher salinities at 5 °C after three and six weeks. At salinities of 0 and 10 ppt, the cotyledon broke through the seed coat in the first week and the mean length of seedlings were 0.56 and 1.22 cm, respectively at three weeks; these increased to 1.72 and 3.8 cm, respectively, at six weeks. However, leaf differentiation had not occurred at 6 weeks. At 20 ppt and 30 ppt, the cotyledon broke through the seed coat in the first week and after three weeks the seedlings elongated to 2.54 and 3.32 cm in length, respectively, and 4.82 and 5.64 cm, respectively, after six weeks. Leaf and adventitious root differentiation occurred at week 6. The highest weight of seedling (25.5 ± 3.5 mg) among all treatments had reached at 30 ppt after six weeks, and ANOVA detected significant differences between the remaining treatments at 5 °C. In the low salinities (0 and 10 ppt), a significantly higher seed germination rate was detected; however, these seedlings had a lower number of leaves, shorter seedling length, and lower WW. This suggested that *Z. marina* seedlings exposed to low salinities exhibited slower development that may reduce their eventual survival over longer exposure times than those tested in the present study. In contrast, the seedling survival rate was greater at higher salinities.

**Table 3:** Ecological morphology of *Zostera marina* L. seeds and seedlings.

Time	Ecological morphology		Salinity 0 ppt	Salinity 10 ppt	Salinity 20 ppt	Salinity 30 ppt
1st week	Germination performance with rate (%)	Day 1	First appearance of germination	First appearance of germination	—	—
		Day 2	2.33 ± 0.58	0.67 ± 0.58	First appearance of germination	—
		Day 4	11.67 ± 0.58	2.00 ± 1.00	0.67 ± 0.58	First appearance of germination
	Other morphological changes		Whole embryo breaks through the seed coat all at once		Cotyledon breaks through the stoma and then the embryo breaks through the seed coat	
3rd week	Germination rate (%)		81.67 ± 10.21^a^	72.67 ± 6.35^a^	25.67 ± 5.86^b^	9.67 ± 4.04^c^
	Seedling length (cm)		0.56 ± 0.21^a^	1.22 ± 0.33^a^	2.54 ± 0.34^b^	3.32 ± 0.28^b^
	Number of leaves/seedling		No leaf	No leaf	First true leaf appears; the leaf is shorter than the cotyledonary blade	
	Other morphological changes		Top of cotyledonary blade becomes brown	Cotyledonary blade becomes longer	Occurrence of adventitious root differentiation	
6th week	Germination rate (%)		85.67 ± 4.93^a^	74.67 ± 7.23^a^	32.33 ± 4.73^b^	11.33 ± 4.51^c^
	Seedling length(cm)		1.72 ± 0.48^a^	3.80 ± 1.10^b^	4.82 ± 0.56^b^^c^	5.64 ± 0.96^c^
	Seedling wet weight/shoot (mg)		8.5 ± 0.5^a^	10.5 ± 2.5^a^	19.5 ± 2.0^b^	25.5 ± 3.5^c^
	Number of leaves/seedling		0 (No leaf)	0 (No leaf)	Second true leaf was observed; the cotyledonary blade is shorter than the leaves	
	Leaf length (cm)		0^a^	0^a^	2.34 ± 0.12^b^	4.48 ± 0.34^c^
	Other morphological changes		The brown part decays continually	Occurrence of leaf differentiation	The cotyledonary blade wizens; the leaves elongates	

**Note:**

Different letters indicate significant differences at p < 0.05 (mean ± standard error).

### Seedling establishment rate for seeds germinated at reduced salinities and transferred to natural seawater (32 ppt)

**Figure 4:**
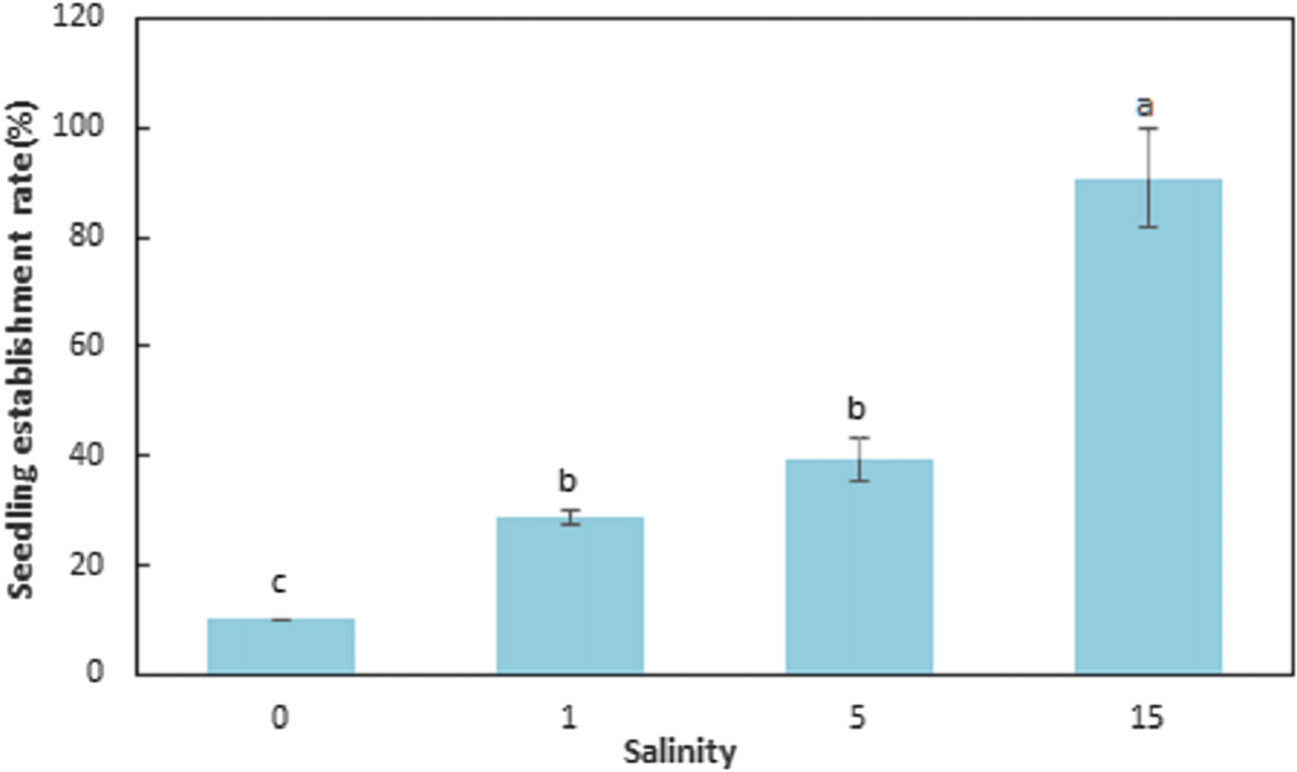
Seedling establishment rate for *Zostera marina* L. seeds germinated at reduced salinities and transferred to natural seawater (32 ppt). Different letters indicate significant differences at p < 0.05 (mean ± standard error).

The seedling establishment differed significantly (F = 143.089, df = 11, p < 0.001) among salinities (Table 4; Fig. 4). Salinity had a large influence on seedling establishment. The SER for seeds germinated at 0, 1, 5, and 15 ppt at 15 °C and then transferred to natural seawater (32 ppt) at 15 °C, were 10.00 ± 0.00, 28.67 ± 1.15, 39.33 ± 4.16, and 90.67 ± 9.02%, respectively. The SER at 15 ppt was significantly higher than the other levels of salinity (F = 61.793, df = 11, p < 0.001) and seedling establishment decreased with decreasing salinities (F = 143.089, df = 11, p < 0.001). Elevated salinity increased seedling establishment at 15 °C (F = 143.089, df = 11, p < 0.001).

**Table 4:** One-way analysis of variance of salinity on the seedling establishment rate of *Zostera marina* L. seeds.

Variable	Df	Sum Sq	Mean Sq	F value	P (> F)
Salinity	3	2,682.92	894.306	143.089	< 0.001
Residuals	8	50	6.25		

**Note:**

Data are not transformed and assumption of homogeneity of variance is met by Levene’s test (P = 0.069).

### Morphological changes in germinated seeds and seedlings

**Figure 5:**
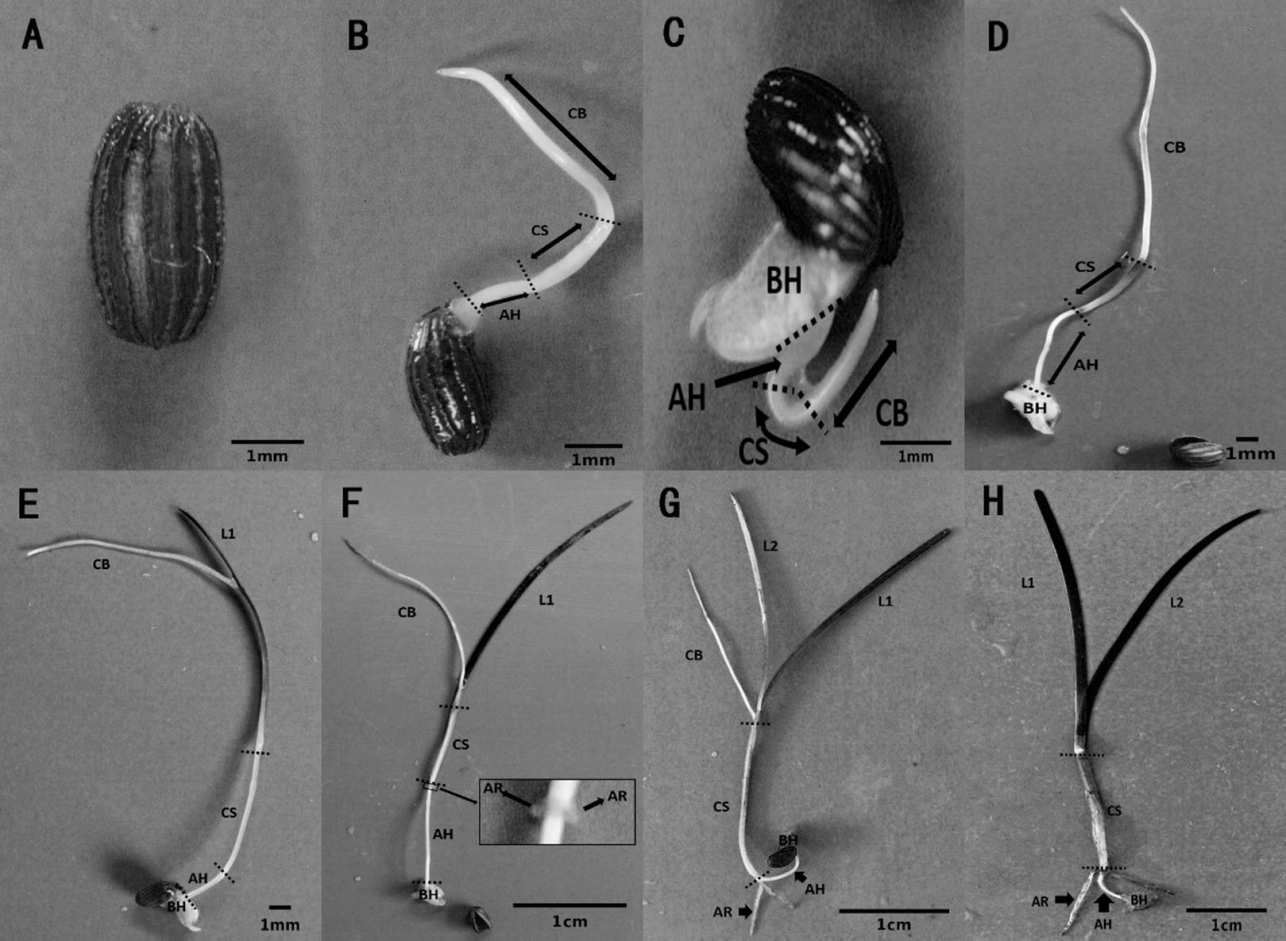
Metamorphosis of *Zostera marina* L. seeds and seedlings during and after germination. (A) Stage 0: a mature seed coated with a dark brown seed coat. (B, C) Stage 1: seed germination. Arrowed lines indicate portions of the sectioned embryo: CB, cotyledonary blade and CS, cotyledonary sheath, respectively; AH, axial hypocotyls and BH, basal hypocotyls, respectively. (D) Stage 2: pre-seedling establishment stage in which germinated seeds have no true leaf or adventitious roots. (E) Stage 3: initial stage of a seedling with first true leaf (L1) differentiation at the base of the cotyledonary blade. (F) Stage 4: seedling with adventitious root (AR) differentiation at the base of the cotyledonary sheath. (G) Stage 5: seedling with second true leaf (L2) differentiation at the base of the cotyledonary blade. (H) Stage 6: intact seedling lacking cotyledonary blade.

The metamorphosis of *Z. marina*, at various growth stages during seed germination and seedling establishment, was observed in the seed germination experiment. Seed germination and seedling growth were divided into seven stages using a method modified method after Sugiura et al. (2009). Stage 0 (Fig. 5A) was defined as the pre-germination stage when the seeds were ripe, plump, and intact. At Stage 1, the seeds broke through the seed coat in one of two ways, either via the cotyledon through the stoma with the embryo then breaking through the seed coat (Fig. 5B), or the whole embryo breaking through the seed coat all at once (Fig. 5C). At Stage 2 (Fig. 5D), the cotyledonary blade (CB), cotyledonary sheath (CS), and axial hypocotyl (AH) of the seedlings were found to be elongate from the central sulcus of basal hypocotyl (BH). At Stage 3 (Fig. 5E), the process of leaf differentiation occurred. The first true leaf (L1) was elongated from the basis of the CS and the first to emerge was the epidermis, followed by the mesophyll, and finally the vein. At Stage 4 (Fig. 5F) adventitious root differentiation occurred. At Stage 5 (Fig. 5G), with the growth of adventitious roots (AR), the second true leaf (L2) emerged from the basis of the CS. At Stage 6 (Fig. 5H), the CB withered away with the growth of the AR and the second true leaf.

## Discussion

### Salinity effects

Studies conducted in laboratory and in the field worldwide have shown that salinity, considered a major factor in seed ecology, has a large influence on seed germination (Caye & Meinesz, 1986; Caye et al., 1992; Conacher et al., 1994; Walmsley & Davy, 1997; Orth et al., 2000; Marion & Orth, 2010). The majority of previous studies for seagrasses have demonstrated that optimal seed germination often occurs under hyposalinity conditions and that germination of most species is reduced at elevated salinities, despite the fact that such low salinity is rarely encountered in the field. For example, Conacher et al. (1994) reported a higher germination rate in *Z. capricorni* seeds at low salinities (< 10 ppt) compared with high salinities (20–40 ppt). *Zostera noltii* seeds were shown to germinate better in low salinity conditions with maximum germination occurring at 1 ppt (Hootsmans, Vermaat & Van Vierssen, 1987); this was supported with the findings of Loques, Caye & Meinesz (1990).

However, other studies have shown little or no effect of salinity on germination. For example, no difference was found in *Z. marina* seed germination between 15 and 35 ppt in the laboratory (McMillan, 1983). Orth & Moore (1983), who studied *Z. marina* seed germination along salinity gradients, also drew a similar conclusion. Salinity stress on germination varies not only among species, but also within a species, with seeds adopting different survival strategies (Kim et al., 2013). Although low salinity has been shown to facilitate germination of *Z. marina* seeds in several populations, no significant differences correlating with salinity have been reported in the Gulf of California (Phillips, Grant & McRoy, 1983). It seems apparent that salinities affect seed germination differently, depending on population.

In this study, we found that freshwater and low salinities (1, 5, 10, and 15 ppt) were able to significantly facilitate *Z. marina* seeds germination. Kaldy et al. (2015) reported the same result in *Z. japonica*. Seed germination in *Z. japonica* was inhibited at salinities of 20 ppt and above, but germination resumed when seeds were placed in freshwater (Kaldy et al., 2015). Hootsmans, Vermaat & Van Vierssen (1987) found that *Z. marina* seeds germinated better under conditions of low salinity (maximum germination occurred at 1 ppt), similar to our results. In our study, we recorded the highest germination rate (88.67 ± 5.77%) of *Z. marina* seeds in freshwater at 15 °C.

Seagrasses began colonizing marine environments from freshwater and terrestrial environments and fully adapted to marine conditions 100 million years ago in the Cretaceous period, establishing in a vast new habitat free of terrestrial competitors and insect pests (Hartog, 1970; Olsen et al., 2016). In the present study, we observed that maximum *Z. marina* seed germination occurred in freshwater and at low salinities, indicating that freshwater and low salinity facilitate seed germination. This might reflect a relic of *Z. marina* evolution or an evolutionary throwback to its previous freshwater existence, common in the early development of various species (Arber, 1920; Chen, Wang & Zhu, 2012).

Given the fact that the germination of some species is significantly reduced at increased salinity or reduced temperature, high salinity and low temperature conditions could be a suitable for the long-term storage of *Z. marina* seeds to maintain germination potential. Kishima, Harada & Sakurai (2011) reported the same result in *Z. japonica*. However, increased salinity may result in toxic ion accumulation within the cell membrane, indirectly inhibiting the activities of some enzymes or causing cell death, thereby influencing seed germination (Katembe, Ungar & Mitchell, 1998).

In addition to its large influence on germination, salinity also plays an important role in seedling establishment and growth. Several studies have found differences in the optimum salinity range between seed and seedling stages, implying that the ability of seagrasses to cope with salinity stress may change during their development (Hootsmans, Vermaat & Van Vierssen, 1987). In our study, germination increased at reduced salinities, as mentioned above. In contrast, the seedling length, weight, number of leaves, and the progress of differentiation were found to be significantly reduced or inhibited at low salinities. For *Posidonia oceanica* seedlings, higher mortality in hyposaline conditions has been observed, while under hypersaline treatments, a lower seedling mortality occurs, and seedling survival is greater at the highest salinities (Fernández-Torquemada & Sánchez-Lizaso, 2013). In the present study, germination of *Z. marina* seeds in freshwater or at low salinities was very high, while very few of the germinated seeds completed leaf differentiation and seedling establishment in freshwater or low salinity, and seedling length was shorter than 4 cm. These results imply that freshwater and low salinity may have a large influence on the establishment and colonization of *Z. marina* in the field and could therefore potentially limit its distribution in coastal and estuarine waters. Our results indicate that the optimum salinity for *Z. marina* seedling establishment and colonization is above 20 ppt in the field, consistent with study conducted by Salo & Pedersen (2014). The optimum salinity for *Z. marina* seedlings in the present study was consistent with that reported for *P. oceanica* by Fernández-Torquemada & Sánchez-Lizaso (2013). The present study indicated that once *Z. marina* seeds germinate, they grow better at high salinities (20–30 ppt) than at low salinities (< 20 ppt).

Our study showed that germination rate of *Z. marina* seeds increased in low salinity. Germination rates in natural seawater were very low (< 15%). In contrast, germination rates of *Z. marina* seeds in freshwater or lower salinities than natural seawater (10 and 20 ppt) were very high (> 80%). However, very few of these seedlings completed leaf differentiation or established in freshwater or the lower salinities (10 and 20 ppt). In contrast, seeds germinated in freshwater or low salinities (1, 5, and 15 ppt) transferred to natural seawater to accomplish seedling establishment achieved high germination rates (10.00, 28.67, 39.33, and 90.67, respectively). Therefore, these may be the optimal incubating conditions to consider when utilizing seeds in the establishment and restoration of seagrasses. After seedlings grow to the stage in which they show their second true leaf, they can be transplanted for seagrass colonization or restoration.

### Temperature effects

Annual temperature plays an important role in controlling site specific seasonal seagrass growth (Lee, Park & Kim, 2007). Zhou et al. (2015) reported that the shoot height of *Z. marina* from the Swanlake Lagoon was positively correlated with temperature. Similar to seagrass growth, seasonal fluctuations in temperature have been shown to control the germination of seagrass seeds from several species (Moore, Orth & Nowak, 1993; Walmsley & Davy, 1997; Probert & Brenchley, 1999; Pan et al., 2011a). It appears that the lowest seawater temperature of the local coastal area is related to the optimal seawater temperature for *Z. marina* seed germination (McMillan, 1983; Orth & Moore, 1983). Seasonal temperature variations have been found to control the timing of germination in a number of seagrass species (Moore, Orth & Nowak, 1993; Taylor, 1957; Conacher et al., 1994). *Zostera marina* has been shown to be adapted to autumn/winter germination in Chesapeake Bay (Moore, Orth & Nowak, 1993), and to winter/spring germination in Prince Edward Sound (Taylor, 1957). Seeds of *Z. capricorni* (Conacher et al., 1994) germinated across a range of temperature treatments (15–30 °C) at low salinities (1, 5, and 10 ppt), while seeds held at higher salinities (20, 30, and 40 ppt) germinated only in lower temperature treatments. Several studies suggest that temperature stratification may be critical for seed germination (Loques, Caye & Meinesz, 1990; Harrison, 1991; Conacher et al., 1994). Brenchley & Probert (1998) found that 16 °C is the optimum temperature for seed germination of *Zostera marina*, with a 100% germination rate in natural seawater. In the present study, we observed that 15 °C was the most suitable temperature for the germination of *Z. marina* seeds with the maximum rate of 88.67 ± 5.77% recorded in freshwater, consistent with the aforementioned studies.

### Implications for restoration

Mature *Z. marina* seeds exhibited dark color with relatively low moisture content and high weight. The mature seeds can be stored at high salinities and low temperature during long-term storage. Seed germination rates increases with increasing temperature and decreasing salinity. Seeds germination can be conducted in low salinities; and the transfer of germinated seeds from freshwater or low salinities to natural seawater can increase SER for restoration of seagrass habitats.

## Conclusion

In conclusion, the germination rates of *Z. marina* seeds in freshwater or low salinities were very high, but none of the seedlings completed leaf differentiation or seedling establishment. However, seeds germinated in freshwater or low salinities could be transferred to natural seawater to accomplish seedling establishment and colonization. Therefore, the transfer of germinated seeds from freshwater or low salinities to natural seawater may be the optimal method in the establishment and restoration of seagrass habitats. In addition, high salinity and low temperatures were suitable for the preservation of *Z. marina* seeds to maintain germination potential. These results may serve as useful data in *Z. marina* habitat restoration.

## Supplemental Information

10.7717/peerj.2697/supp-1 Supplemental Information 1 Raw data: Germination rates, ecological morphology and seedling establishment rate of *Zostera marina* L. seeds subjected to different salinities and temperatures.

## Additional Information and Declarations

### Field Study Permissions

The following information was supplied relating to field study approvals (i.e., approving body and any reference numbers): The collecting of the reproductive shoots of Z. marina from Swan Lake of Weihai was permitted by Peiliang Wang, manager of Mashan Group Co. Ltd. Ethical approval was not required for this study because no endangered animals and plants were involved. However, specimen collection and maintenance were performed in strict accordance with the recommendations of China Society of Plant Protection.

### Data Deposition

The following information was supplied regarding data availability: The raw data has been supplied as Supplemental Dataset Files.
